# Berberine: A Rising Star in the Management of Type 2 Diabetes—Novel Insights into Its Anti-Inflammatory, Metabolic, and Epigenetic Mechanisms

**DOI:** 10.3390/ph18121890

**Published:** 2025-12-14

**Authors:** Da Liu, Liting Zhao, Ying Wang, Lei Wang, Donglu Wu, Yangyang Liu

**Affiliations:** 1 School of Pharmacy, Changchun University of Chinese Medicine, Changchun 130117, China; liuda@ccucm.edu.cn (D.L.); 23203089313@stu.ccucm.edu.cn (L.Z.); 23203560022@stu.ccucm.edu.cn (Y.W.); 13578702468@163.com (L.W.); 2 Public Experimental Center, Changchun University of Chinese Medicine, Changchun 130117, China; 3 School of Clinical Medical, Changchun University of Chinese Medicine, Changchun 130117, China; 4 College of Traditional Chinese Medicine, Changchun University of Chinese Medicine, Changchun 130117, China

**Keywords:** berberine, type 2 diabetes mellitus, anti-inflammation, insulin resistance, glucose metabolism, drug combination

## Abstract

Type 2 diabetes mellitus (T2DM) is a widespread metabolic disorder characterized by insulin resistance and pancreatic β-cell dysfunction, posing a substantial global health challenge. This review systematically summarizes the therapeutic potential of berberine, a natural isoquinoline alkaloid, in the management of T2DM. Berberine’s pharmacological activities are discussed from multiple perspectives, including enhancing insulin sensitivity and regulating glucose metabolism—encompassing glycogen synthesis, gluconeogenesis, and glucose transport. The review also highlights berberine’s anti-inflammatory, antioxidant, and epigenetic enzyme-targeting actions and its involvement in key T2DM-related signaling pathways such as AKT, AMPK, and GLUTs. These findings collectively elucidate the multi-targeted and multi-pathway molecular mechanisms underlying berberine’s efficacy against T2DM. Additionally, the review covers the pharmacological activities and molecular mechanisms of berberine in treating T2DM complications—including diabetic nephropathy, retinopathy, cardiomyopathy, neuropathy, and diabetic foot ulcers—as well as its clinical and preclinical applications and the synergistic benefits of combination therapy with agents such as metformin, ginsenoside Rb1, and probiotics. By systematically reviewing the literature retrieved from PubMed and Web of Science up to 2025, this article provides a comprehensive summary of current research, offering a theoretical foundation for the clinical use of berberine in T2DM therapy.

## 1. Introduction

Diabetes is a metabolic disorder characterized by chronic hyperglycemia resulting from inadequate insulin action, which is classified into several forms, including type 1 diabetes, type 2 diabetes (T2DM), gestational diabetes, and other rare types [1]. T2DM accounts for more than 90% of all diabetes cases globally, and its prevalence continues to rise annually, with an observed trend toward onset in younger populations [2]. By 2045, over 700 million individuals will be affected by T2DM worldwide [3]. The pathogenesis of T2DM is multifactorial, primarily involving pancreatic β-cell dysfunction and insulin resistance in peripheral tissues, which collectively contribute to disrupted glucose homeostasis and chronic low-grade inflammation [4]. Sustained hyperglycemia promotes glucotoxicity, significantly increasing the risk of multi-organ damage and diabetic complications [5,6,7]. These encompass microvascular impairments—such as retinopathy, nephropathy, and neuropathy—as well as macrovascular conditions like atherosclerotic ischemia [5].

Current management of T2DM includes lifestyle interventions such as a balanced diet and regular exercise, along with pharmacological approaches involving oral hypoglycemic agents and subcutaneous insulin injections [1,4]. While conventional treatments like metformin and insulin remain widely used, several novel glucose-lowering drugs—including glucagon-like peptide-1 (GLP-1) receptor agonists and sodium-glucose co-transporter-2 (SGLT2) inhibitors—have been introduced into clinical practice in recent years [1,8,9]. In parallel, natural compounds have garnered increasing attention as potential therapeutic options due to their favorable safety profiles, minimal side effects, and well-documented efficacy, a number of these compounds exhibit notable antidiabetic properties [9,10,11]. For instance, resveratrol has been shown to improve insulin resistance and reduce fasting blood glucose in patients with T2DM [12,13]. Similarly, anthocyanins from black bean seeds can activate AMPK and GLUT4-related pathways, ameliorate hyperglycemia, and restore insulin sensitivity in murine models [14,15].

**Figure 1:**
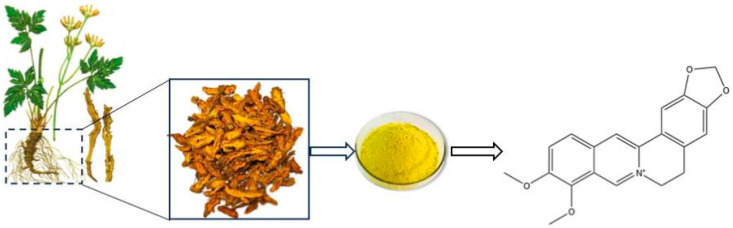
Berberine from *Coptis chinensis*: Botanical Source and Chemical Structure. This flowchart illustrates the complete process of selecting a specific part (the root, indicated by the dotted box) from the whole plant, processing it into a directly usable raw material (the dried rhizome, indicated by the solid box), physically transforming it into powder, and finally isolating the target chemical compound from it. The arrows clearly indicate the transformation relationship of each step.

Berberine, an isoquinoline alkaloid derived from plants such as *Coptis chinensis* (Figure 1), exhibits a broad spectrum of pharmacological activities [16]. Which include antihyperglycemic, antihyperlipidemic, antibacterial, anti-inflammatory, antioxidant, gut microbiota-modulating, anticancer, and immunomodulatory effects [17,18]. Recent studies have further elucidated its mechanisms in diabetes treatment, such as promoting insulin secretion, alleviating insulin resistance, inhibiting gluconeogenesis, enhancing glucose uptake and glycolysis, mitigating inflammation and oxidative stress, and modulating gut microbiota composition [18,19,20]. Moreover, the beneficial effects of berberine on lipid metabolism, cardiovascular function, and neuroprotection indicate its potential in preventing and treating T2DM complications were also revealed [17]. Focusing on insulin signaling and epigenetic regulation, this review summarizes the pharmacological roles and molecular mechanisms of berberine in the management of T2DM and its complications, and discusses its prospects for clinical translation.

To systematically evaluate the existing evidence on berberine, we conducted a comprehensive literature search in PubMed and Web of Science, with the search period extending up to 2025. The search terms included “berberine,” “type 2 diabetes,” “diabetic complications,” “insulin resistance,” and other relevant keywords. This review summarizes the included studies from seven key perspectives: berberine’s regulation of glucose metabolism, glucose transport, insulin signaling pathways, epigenetic modifications, anti-inflammatory and antioxidant effects, protection of pancreatic β-cells, promotion of insulin secretion, and induction of glucagon-like peptide-1 (GLP-1) secretion. We systematically outline the pharmacological activities and molecular mechanisms of berberine in the management of T2DM and its complications, and further discuss its prospects for clinical translation.

## 2. Role of Berberine in T2DM Therapy

### 2.1. Regulation of Glucose Metabolism by Berberine

Carbohydrates serve as essential energy sources for the body, and their metabolic homeostasis is critical for maintaining normal blood glucose levels and sustaining energy supply across tissues [21,22]. In T2DM, dysregulated carbohydrate metabolism leads to persistently elevated blood glucose, which contributes to the development of various complications [5].

Glycogen synthesis—the biochemical process by which glucose is converted into glycogen and stored in tissues such as the liver and muscle—plays a vital role in glucose homeostasis and energy reserve maintenance [23,24,25]. Studies have demonstrated that berberine enhances hepatic glycogen synthesis both in vivo and in vitro, as observed in palmitic acid- and dexamethasone-treated HepG2 cells, liver tissues of db/db mice, and streptozotocin-induced diabetic C57BL/6 mice [26,27,28,29,30]. Glycogen synthase kinase 3 beta (GSK3β), a widely expressed serine/threonine protein kinase, inhibits glycogen synthesis by phosphorylating and inactivating glycogen synthase (GS) [31,32]. Berberine has been shown to promote the phosphorylation of GSK3β via modulation of relevant signaling pathways, thereby enhancing glycogen synthesis [33]. In fructose-induced diabetic mice, berberine increased GSK3β phosphorylation and stimulated hepatic glycogen deposition [34]. Similar effects were observed in streptozotocin-induced and high-fat diet (HFD)-fed diabetic mice, where berberine upregulated GSK3β phosphorylation and raised hepatic glycogen content [30,35]. Hepatic glucokinase (GK) is a key enzyme in glucose metabolism, which initiates the glycogen synthesis pathway by catalyzing the phosphorylation of glucose to produce glucose-6-phosphate, thereby supplying the essential substrate for glycogen synthesis [36,37]. Berberine treatment increased GK expression and glycogen levels in high-glucose-induced insulin-resistant AML12 and HuH7 hepatocyte models [14,15]. Consistent with these findings, upregulation of GK expression and enhanced glycogen synthesis were also detected in the liver tissues of db/db mice and islet tissues of Sprague Dawley (SD) rats following berberine administration [38,39].

Gluconeogenesis—the metabolic process through which non-carbohydrate precursors such as lactate, glycerol, and glucogenic amino acids are converted into glucose—is often abnormally enhanced in T2DM, contributing to hyperglycemia. Phosphoenolpyruvate carboxykinase (PEPCK) and glucose-6-phosphatase (G6Pase) are key rate-limiting enzymes in this pathway [40,41]. In db/db mice, hepatic protein levels of PEPCK and G6Pase have been shown to be significantly elevated compared with those in normal controls [42]. Accumulating evidence indicates that berberine downregulates the mRNA expression of PEPCK and G6Pase in various insulin-resistant models, including liver tissues of C57BL/6J mice, dexamethasone-induced diabetic mice, ob/ob mice, and HepG2 insulin-resistant cells, thereby suppressing gluconeogenesis [28,43,44]. The transcription factor forkhead box protein O1 (FOXO1) plays a pivotal role in cellular metabolism and promotes the expression of PEPCK and G6Pase to stimulate gluconeogenesis [45,46]. Under T2DM conditions, impaired insulin signaling leads to inadequate suppression of FOXO1 activity, resulting in excessive hepatic glucose output [47]. In insulin-resistant HepG2 cells, FOXO1 upregulation correlates with increased PEPCK and G6Pase levels and enhanced glucose production [45,48]. Berberine treatment has been shown to reduce FOXO1 protein levels along with PEPCK and G6Pase expression in the livers of high-fat diet-induced diabetic rats [49]. Moreover, berberine can directly bind to and inhibit FOXO1, thereby attenuating FOXO1-mediated gluconeogenesis [50,51,52]. Beyond FOXO1, berberine also modulates other transcriptional regulators of gluconeogenesis. In high-fat diet-induced diabetic mice, berberine suppresses the HNF-4α-mediated upregulation of PEPCK and G6Pase in the liver [53]. A similar coordinated downregulation of HNF-4α, PEPCK, and G6Pase protein levels was observed in db/db mice following berberine administration [42]. Additionally, the cAMP response element-binding protein (CREB) is an important transcriptional activator of gluconeogenic genes [54]. In diabetic states, glucagon stimulates CREB phosphorylation via the cAMP pathway, enhancing the expression of G6Pase and PEPCK [55,56]. Studies have shown that in ob/ob mouse hepatocytes and liver tissues, berberine facilitates cAMP degradation, thereby inhibiting CREB phosphorylation and subsequently reducing Pepck and G6pase mRNA levels [44]. Consistent with this, berberine treatment in primary rat hepatocytes led to decreased phosphorylation of CREB and reduced protein expression of Pepck and G6pase [57].

### 2.2. Regulation of Glucose Transport by Berberine

The transmembrane transport of glucose, a vital process for cellular glucose uptake and utilization in eukaryotes, is predominantly mediated by the glucose transporter (GLUT) family [58]. Comprising 14 distinct isoforms, this family plays a fundamental role in the maintenance of systemic glucose homeostasis [59]. In insulin-sensitive tissues, glucose entry is facilitated by GLUT proteins, which are essential for blood glucose regulation [60]. In type 2 diabetes, however, both the expression and functional activity of GLUTs are diminished, leading to impaired cellular glucose uptake and consequent hyperglycemia [60,61].

GLUT1 is the most ubiquitously expressed glucose transporter isoform in the human body, present in nearly all tissues [62,63]. The dysfunction of GLUT1 can compromise cellular glucose transport efficiency and impair systemic glucose regulation. Studies have shown that berberine upregulates GLUT1 expression in L929 fibroblasts and enhances GLUT1-mediated glucose uptake [62]. Similarly, in 3T3-L1 cells, berberine induces phosphorylation of ERK and AMPK, leading to increased GLUT1 protein expression and subsequent potentiation of glucose uptake [59].

GLUT2 is primarily expressed in organs involved in systemic glucose release, such as the liver, intestine, and pancreatic β-cells [61,64]. Under insulin-resistant conditions, impaired insulin signaling reduces GLUT2 transport activity, diminishing glucose uptake in these tissues and contributing to hyperglycemia [65]. Berberine has been demonstrated to enhance GLUT2 expression via the PPARγ-FGF21 pathway in the livers of high-fat diet/streptozotocin-induced diabetic mice and in glucosamine-induced HepG2 cells, thereby promoting glucose uptake [65]. Additionally, berberine upregulates GLUT2 expression in high-fat diet-induced diabetic mice and oleic acid-treated HepG2 cells, improving glucose uptake and mitigating insulin resistance [66]. In the intestine, glucose absorption is largely mediated by GLUT2 in epithelial cells [67]. Postprandially, high luminal glucose levels trigger rapid translocation of GLUT2 to the brush border membrane, facilitating dietary glucose uptake via facilitated diffusion [68]. Berberine has been found to suppress IGF-1R phosphorylation-mediated GLUT2 translocation in IEC-6 intestinal epithelial cells, thereby reducing intestinal glucose absorption [69].

GLUT4 is an insulin-sensitive glucose transporter predominantly expressed in skeletal muscle, cardiac muscle, and adipose tissue [70]. Under physiological conditions, insulin stimulates the translocation of GLUT4 from intracellular vesicles to the plasma membrane, thereby enhancing cellular glucose uptake [61]. In type 2 diabetes, however, insulin resistance or deficient insulin secretion impairs GLUT4 translocation, leading to diminished glucose uptake and consequent hyperglycemia [71,72,73]. Studies have shown that berberine can upregulate GLUT4 expression in both skeletal and cardiac muscle tissues. For instance, in diabetic rat models, berberine elevates GLUT4 mRNA and protein levels in skeletal muscle [74]. Similarly, in palmitic acid-induced insulin-resistant H9c2 cardiomyocytes, berberine enhances AKT activation, thereby increasing GLUT4 expression and promoting glucose uptake [75]. Beyond the AKT pathway, berberine also modulates GLUT4 through nuclear receptor signaling. Molecular docking analyses indicate that berberine exhibits binding affinity for both PPARα and PPARγ, nuclear receptors involved in glucose and lipid metabolism [76,77]. In insulin-resistant HepG2 cells, berberine upregulates the mRNA expression of PPARα, PPARγ, and GLUT4, thereby enhancing glucose uptake. This effect is attenuated when PPAR inhibitors are applied, confirming the involvement of PPAR signaling in berberine-mediated GLUT4 regulation [77]. Additionally, in skeletal muscle of high-fat diet-induced mice and in C2C12 myotube models, berberine downregulates miR-27a expression, leading to increased phosphorylation of IRS1 and AKT, elevated GLUT4 levels, and enhanced glucose uptake [78].

### 2.3. Regulation of the Insulin Signaling Pathway by Berberine

**Figure 2:**
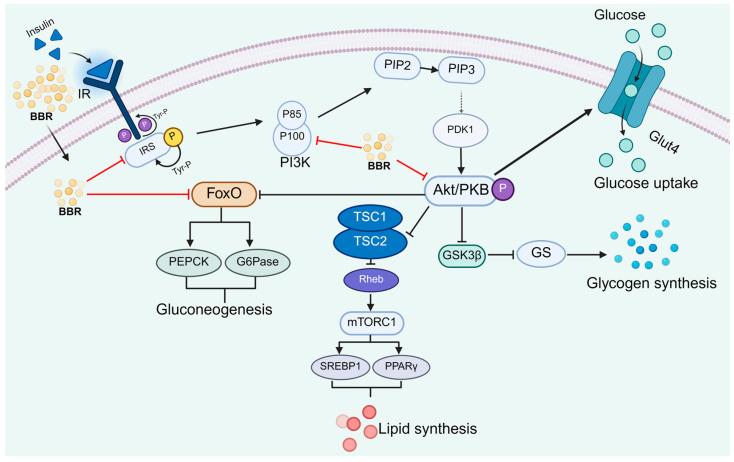
Berberine Ameliorates Insulin Resistance by Modulating the Insulin Signaling Pathway and Key Metabolic Nodes. Created in BioRender. tt, z. (2025) https://BioRender.com/6pp3j10 (accessed on 11 December 2025). The arrows in the figure indicate the nature of regulation: solid black arrows represent positive regulation (activation/promotion), while black flat-headed arrows represent negative regulation (inhibition/blocking). Red flat-headed arrows specifically indicate the inhibitory effects of berberine (BBR).

Insulin resistance (IR) refers to a pathological state in which peripheral tissues and target organs exhibit diminished sensitivity to insulin, leading to impaired glucose uptake and utilization, and consequently contributing to hyperglycemia [79,80]. The insulin signaling pathway serves as a critical intracellular cascade for regulating glucose and lipid metabolism [81,82]. Upon insulin binding, the insulin receptor (IR) on the cell surface activates its intrinsic tyrosine kinase activity, resulting in autophosphorylation and subsequent recruitment and phosphorylation of insulin receptor substrate (IRS) proteins, such as IRS-1 [83,84]. This initiates a downstream signaling cascade that promotes cellular glucose uptake and fatty acid utilization, thereby maintaining glucose and lipid homeostasis [85]. Studies have demonstrated that berberine enhances cellular insulin sensitivity and ameliorates insulin resistance by modulating key components of the insulin signaling pathway—including InsR, IRS-1, AKT, and AMPK (Figure 2).

AKT (protein kinase B, PKB) serves as a central effector in the insulin signaling pathway, by modulating downstream targets including GSK3β and FOXO1, AKT facilitates glucose uptake, promotes glycogen synthesis, and suppresses gluconeogenesis, thereby playing an essential role in glucose homeostasis [86]. Dysregulation of AKT signaling is frequently observed in T2DM, contributing to the development of insulin resistance (IR) [87]. Under insulin-resistant conditions, AKT activation is compromised [88]. For instance, both in the liver tissues of high-fat diet (HFD)-fed mice and in insulin-resistant HepG2 cellular models, AKT expression is notably downregulated [89].

Berberine has been demonstrated to enhance AKT phosphorylation across multiple experimental systems. In vivo and in vitro studies indicate that berberine upregulates phosphorylated AKT levels in the hippocampus of chronically restrained mice, in cardiac tissue of lipopolysaccharide (LPS)-induced septic mice, and in rotenone-treated SH-SY5Y cells [90,91,92]. Furthermore, berberine promotes AKT activation by modulating upstream regulators such as IRS-1 and PI3K, which in turn facilitates downstream signaling and ameliorates insulin resistance [93,94].

Insulin receptor substrate 1 (IRS-1) is a critical mediator of insulin signal transduction in insulin-sensitive tissues [83]. In an HFD-induced gestational diabetes rat model, berberine increased serine phosphorylation of IRS-1 in hepatic tissue, elevated the p-AKT/AKT ratio, and improved fasting blood glucose and insulin resistance indices [95]. Similarly, in insulin-resistant primary hepatocytes, berberine enhanced IRS-1 serine phosphorylation while reducing tyrosine phosphorylation, leading to increased AKT activation [96].

Phosphoinositide 3-kinase (PI3K) catalyzes the production of phosphatidylinositol-3-phosphate (PIP3), which subsequently activates AKT and serves as a key node in intracellular signaling [97]. Berberine was shown to activate PI3K and stimulate AKT phosphorylation in ovarian tissues from a polycystic ovary syndrome (PCOS) rat model [97,98]. In the hippocampus of T2D rats, berberine upregulated PI3K-dependent AKT and GSK-3β phosphorylation, significantly reducing blood glucose and serum insulin levels [99]. Moreover, in insulin-resistant HepG2 cells and PCOS rat models, berberine enhanced PI3K kinase activity, leading to AKT-mediated upregulation of GLUT4 expression [93,98].

Fibroblast growth factor 21 (FGF21), a major metabolic regulator, also promotes AKT phosphorylation [100,101]. Berberine upregulates FGF21 expression in HepG2 cells, C2C12 myotubes, and the liver of C57BL/6 mice [65,100,102]. In a glucosamine hydrochloride (Glcn)-induced insulin-resistant HepG2 model, berberine increased FGF21 expression, which subsequently enhanced the phosphorylation of both AKT and GSK3β [65].

Activated AKT modulates downstream effectors such as GSK3β and GLUT4 to alleviate insulin resistance [103]. In HFD-induced diabetic mice, berberine enhanced AKT phosphorylation, leading to GSK3β-mediated glycogen synthesis in the liver [35]. A similar upregulation of phosphorylated AKT and GSK3β was observed in the liver of fructose-induced diabetic mice following berberine treatment [34].

Beyond the AKT pathway, berberine also ameliorates insulin resistance through AMP-activated protein kinase (AMPK), a key cellular energy sensor [104]. AMPK activation regulates hepatic gluconeogenesis and glucose uptake, thereby improving insulin sensitivity [101,105]. In skeletal muscle and adipose tissue of diabetic rats, berberine enhanced LKB1-mediated AMPK phosphorylation, which reduced TORC2 phosphorylation, suppressed PEPCK and G6Pase expression, and lowered fasting blood glucose [106,107]. Similarly, in the liver of fructose-fed mice, berberine increased LKB1 expression and AMPK phosphorylation, improving glucose tolerance and insulin resistance [34]. In insulin-resistant H9C2 cardiomyocytes, berberine promoted AMPK phosphorylation, elevated GLUT4 protein levels, and enhanced glucose uptake, further alleviating insulin resistance [108].

### 2.4. Berberine Induces the Secretion of Glucagon-like Peptide-1 (GLP-1)

Glucagon-like peptide-1 (GLP-1) is an incretin hormone secreted by intestinal L cells. It stimulates insulin secretion in a glucose-dependent manner, inhibits glucagon release, delays gastric emptying, and increases satiety. GLP-1 plays a central role in glucose homeostasis and is an important therapeutic target for type 2 diabetes [109,110]. In patients with T2DM, GLP-1 secretion is reduced and the activity of its receptor signaling path-way is diminished, resulting in insufficient stimulation of insulin secretion and progressive decline of β-cell function [111,112]. Meanwhile, the inhibitory effect of GLP-1 on α cells is weakened, leading to abnormally elevated glucagon secretion and increased hepatic glucose output [113,114]. GLP-1 receptor agonists can improve hyperglycemia [115]. Berberine can induce GLP-1 secretion through various pathways, thereby exerting its therapeutic effects on type 2 diabetes; for example, berberine can specifically activate the bitter taste receptor TAS2R38 in intestinal enteroendocrine STC-1 cells, relying on the phospho-lipase C (PLC) signaling pathway, thereby stimulating the secretion of glucagon-like peptide-1 (GLP-1), which in turn promotes insulin secretion to improve type 2 diabetes [116]. Other studies indicate that berberine can act on mitochondria, inhibit excessive ATP production, thereby improving mitochondrial function to restore L-cell secretory function [117]. Other studies have indicated that the metabolites of berberine, which are locally enriched in the intestine after oral administration (berberrubine and palmatine), can reduce oxidative stress and mitochondrial dysfunction, reverse the inhibition of the Akt pathway caused by inflammation, and enhance GLP-1 synthesis and secretion [118]. Additionally, berberine can reshape the gut micro-biome, promoting the proliferation of short-chain fatty acid (SCFA)-producing bacteria, and subsequently stimulating GLP-1 secretion by activating the GPR41 and GPR43 receptors on L cells, thereby indirectly enhancing the secretion of GLP-1 [110,117]. It is not difficult to understand that the induction of GLP-1 secretion by berberine is critical for maintaining blood glucose stability.

### 2.5. The Anti-Inflammatory and Antioxidant Effects of Berberine

**Figure 3:**
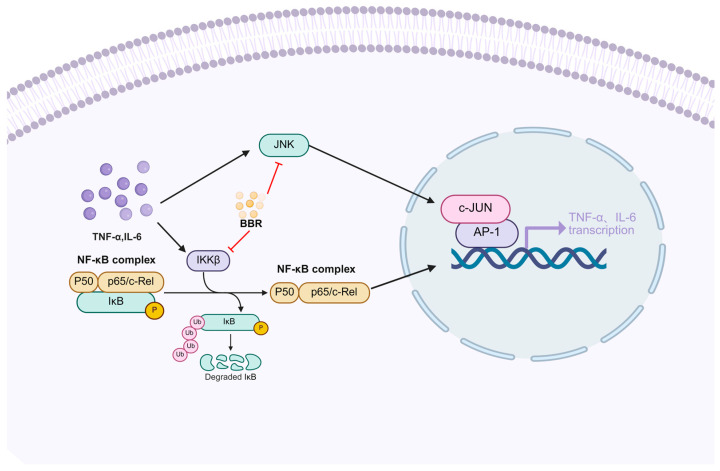
Berberine Inhibits Key Inflammatory Pathways and Reduces the Production of Pro-inflammatory Cytokines. Created in BioRender. tt, z. (2025) https://BioRender.com/paj2rq1 (accessed on 11 December 2025). The arrows in the figure indicate the nature of regulation: solid black arrows represent positive regulation (activation/promotion), while black flat-headed arrows represent negative regulation (inhibition/blocking). Red flat-headed arrows specifically indicate the inhibitory effects of berberine (BBR).

Patients with T2DM exhibit immune system dysregulation, which contributes to a state of chronic inflammation [119,120,121]. Inflammation is a defensive response to various injurious stimuli, in which the immune system plays a critical regulatory role [122]. Chronic low-grade inflammation represents a key pathological feature of type 2 diabetes, commonly characterized by elevated pro-inflammatory cytokines, immune cell imbalance, and gut microbiota dysbiosis [123]. Berberine has been demonstrated the activity of modulating immune responses through multiple pathways, thereby attenuating inflammatory reactions and contributing to the treatment of type 2 diabetes (Figure 3).

It is reported that berberine suppressed inflammatory signaling pathways, leading to reduced expression of pro-inflammatory cytokines [124,125,126,127]. For example, in insulin-resistant HepG2 cells, berberine upregulates PPM1B expression, decreases IKKβ phosphorylation, and inhibits the NF-κB pathway, resulting in downregulated mRNA levels of TNF-α, IL-1β, IL-6, and IL-8, along with upregulated IL-10 expression [93]. In vivo studies further support these findings: in gestational diabetes mellitus (GDM) rat models, berberine suppresses IKKβ phosphorylation and nuclear translocation of NF-κB p65 in liver tissue, thereby reducing TNF-α levels in both serum and liver [95]. Moreover, in insulin-resistant hepatocytes, berberine downregulates MEK1/2 and ERK1/2 phosphorylation, which subsequently influences NF-κB signaling and inhibits the production of TNF-α and IL-6 [96]. In summary, berberine alleviates inflammation under insulin-resistant conditions by modulating inflammatory signaling cascades, enhancing anti-inflammatory cytokine production, and suppressing pro-inflammatory mediators.

Berberine also exerts anti-inflammatory effects through direct suppression of inflammatory cytokine expression [128,129]. In type 2 diabetes, increased macrophage infiltration into adipose tissue, liver, and muscle contributes to the excessive release of pro-inflammatory cytokines—such as TNF-α, IL-6, and IL-1β—which interfere with insulin signaling by impairing processes such as IRS-1 phosphorylation, ultimately promoting insulin resistance [130,131,132]. Studies have shown that in palmitic acid-induced HepG2 cells, berberine suppresses the production of IL-6 and TNF-α in a concentration-dependent manner and restores insulin sensitivity by attenuating inflammation [18]. Consistent with these findings, berberine significantly reduces circulating and vascular smooth muscle levels of IL-6 and TNF-α in HFD/STZ-induced type 2 diabetic rats, further confirming its cytokine-targeted anti-inflammatory activity [133].

### 2.6. Berberine Ameliorates Type 2 Diabetes Through Multi-Target Epigenetic Regulation

Epigenetics involves heritable changes in gene expression that occur without alterations in the DNA sequence, primarily mediated through DNA methylation, histone modifications, and non-coding RNA regulation [134,135]. The pathogenesis of T2DM is influenced not only by genetic factors but also closely associated with epigenetic mechanisms [136,137,138]. Environmental factors, such as high-sugar and high-fat diets, have been shown to induce “metabolic memory” via epigenetic modifications, contributing to persistent metabolic dysregulation [139]. As a natural alkaloid, berberine has emerged as a multi-target epigenetic modulator with therapeutic potential in T2DM [140].

DNA methylation entails the addition of a methyl group to CpG sites, catalyzed mainly by DNA methyltransferases (DNMTs), while demethylation is facilitated by TET family proteins [141]. Aberrant methylation at specific CpG sites in pancreatic islets of T2DM patients has been linked to impaired insulin secretion and aggravated insulin resistance [142]. Berberine counteracts this dysregulation by inhibiting DNMT1 and DNMT3a expression in the kidney tissues of diabetic nephropathy mouse models, thereby preventing methylation of the KLF4 promoter, enhancing KLF4 transcription, and ameliorating renal injury [143]. In dexamethasone-induced insulin-resistant 3T3-L1 adipocytes, berberine reduces HIF3A methylation, leading to increased expression of IRS-1 and GLUT4, improved glucose utilization, and restored insulin sensitivity [144].

Histone acetylation, which involves the addition of acetyl groups to lysine residues on histones, alters chromatin structure and modulates transcriptional activity [141]. Berberine has been reported to regulate class III histone deacetylases (SIRTs) [27,145,146]. In primary mouse hepatocytes, it suppresses SIRT3-mediated deacetylation of PEPCK1, thereby curbing gluconeogenesis [146]. Berberine also modulates SIRT1 activity: in 3T3-L1 adipocytes and adipose tissue of high-fat diet-induced obese mice, it activates the SIRT1-AMPK axis and enhances AKT phosphorylation, collectively improving insulin sensitivity [147]. Similarly, in palmitate-induced HepG2 cells and db/db mouse livers, berberine upregulates SIRT1, activates Opa1, and restores insulin signaling [145]. Furthermore, it suppresses palmitate-induced miR-146b expression in HepG2 cells, leading to SIRT1 upregulation, enhanced FOXO1 deacetylation, promoted glycogen synthesis, and alleviated hepatic insulin resistance [27].

### 2.7. Protective Effects of Berberine on Pancreatic β-Cells and Promotion of Insulin Secretion

The pancreatic islets of Langerhans consist of clustered endocrine cells—including α, β, δ, and PP cells—embedded within the pancreas [148]. Among these, pancreatic β-cells play a central role in glucose homeostasis by secreting insulin to promote glucose utilization [149]. In type 2 diabetes, prolonged hyperglycemia and other metabolic disturbances contribute to pancreatic β-cell damage, resulting in decreased β-cell mass and functional impairment [150,151,152].

Recent studies have demonstrated the protective effects of berberine on pancreatic β-cells in both in vivo and in vitro. For example, in Goto-Kakizaki (GK) rats—a well-established type 2 diabetes model—berberine treatment increased β-cell number and induced morphological improvements, such as larger and more rounded nuclei, ameliorating pancreatic pathology, which were associated with reduced fasting blood glucose and improved insulin sensitivity [153]. In vitro, berberine enhanced β-cell viability and potentiated insulin secretion across multiple cellular models, including palmitic acid-treated MIN6 and INS-1 cells, as well as streptozotocin-induced primary mouse islets [154,155,156,157,158].

Analysis of islet tissue mRNA from HFD/STZ-induced type 2 diabetic mice revealed that berberine downregulates miR-204 and upregulates SIRT1 expression, further reduced fasting blood glucose, triglyceride (TG), and total cholesterol (TC), along with increased HDL-C and fasting insulin (FINS) levels. Berberine also ameliorated pathological alterations in islet morphology, improved insulin secretion, and alleviated insulin resistance. The miR-204–SIRT1 axis was further validated in palmitic acid-injured MIN6 cells, confirming its role in berberine-mediated islet protection [154]. In Rin-5f pancreatic β-cells, berberine enhanced proliferation and upregulated PARP-1 protein expression. Consistently, in HFD/STZ-induced diabetic rats, berberine attenuated the loss of islet cells and elevated insulin levels, suggesting that PARP-1 upregulation contributes to its β-cell-preserving effects [159]. Berberine also mitigates β-cell apoptosis and promotes insulin secretion through the iPLA2β/CL/Opa1 pathway [158]. In palmitic acid-treated MIN6 cells and db/db mouse pancreatic tissue, berberine suppressed the expression of caspase-3 and cytochrome c, exerting anti-apoptotic effects. In vivo, it increased islet area, reduced inflammatory infiltration and vacuolization, and enhanced insulin secretion [158].

Notably, berberine exhibits a glucose-dependent insulinotropic effect. In isolated C57BL/6J mouse islets perfused with high glucose (25 mM), berberine stimulated insulin secretion, whereas no effect was observed under low glucose (2.8 mM) conditions. Similarly, in HFD-fed C57BL/6J mice, berberine lowered blood glucose and elevated serum insulin. A randomized, double-blind, placebo-controlled, two-period crossover, single-dose, phase 1 clinical trial (NCT03972215) demonstrate that berberine enhances glucose-stimulated insulin secretion in humans without altering basal insulin levels [160].

## 3. Mechanisms and Effects of Berberine Combination Therapy

As a natural compound with multi-target pharmacological activities, berberine exhibits considerable potential in the treatment of T2DM, as summarized in previous sections [161]. Accumulating evidence indicates that berberine can be effectively combined with other drugs, resulting not only in improved glycemic control but also in enhanced anti-inflammatory efficacy and gut microbiota regulation, thereby producing superior therapeutic outcomes (Table 1).

Metformin, a first-line medication for T2DM, acts primarily by suppressing hepatic gluconeogenesis and promoting glucose uptake in peripheral tissues [162]. Studies have shown that the combination of berberine and metformin yields synergistic hypoglycemic effects [163]. In db/db mice, the combined treatment led to more pronounced changes in gut microbiota composition compared with either monotherapy, accompanied by further reductions in blood glucose and enhanced insulin sensitivity [164].

Ginsenoside Rb1, a protopanaxadiol-type saponin belonging to the dammarane triterpenoid family, is found in a variety of natural plants and exhibits broad pharmacological properties, demonstrating notable therapeutic potential in the central nervous, cardiovascular, and immune systems [165]. Modern research has revealed that ginsenoside Rb1 significantly attenuates insulin resistance [166]. In insulin-resistant adipocyte models, the combination of berberine and ginsenoside Rb1 synergistically suppressed the NF-κB signaling pathway and alleviated cellular inflammation [167]. Furthermore, in HFD/STZ-induced diabetic rats, co-administration of berberine and ginsenoside Rb1 markedly improved glucose metabolism and insulin sensitivity, leading to reduced blood glucose levels [168]. A similar synergistic effect was observed in db/db mouse models [169].

In clinical settings, the PREMOTE study demonstrated that supplementing berberine with probiotics (Prob) resulted in a greater improvement in the insulin resistance index among patients with type 2 diabetes compared to berberine monotherapy. The group receiving a combination of Prob and berberine also achieved a more pronounced reduction in glycated hemoglobin (HbA1c) than the Prob-alone group, indicating enhanced glucose-lowering efficacy with the combination regimen [20]. Additionally, studies have shown that Prob combined with berberine is superior to either berberine or Prob alone in improving postprandial total cholesterol (pTC) and low-density lipoprotein cholesterol (pLDL-c) levels, which combination therapy effectively mitigates postprandial dyslipidemia in individuals with type 2 diabetes [170].

Numerous studies have demonstrated the beneficial metabolic effects of prebiotics in diabetes [171]. Stachyose (Sta), a prebiotic oligosaccharide, has been shown to improve glycemic control and modulate gut microbiota composition. In HFD-induced diabetic mouse models, the combination of berberine and Sta significantly reduced glycated hemoglobin (HbA1c) levels and the area under the curve (AUC) for blood glucose in both oral glucose tolerance tests (OGTT) and insulin tolerance tests (ITT). Moreover, the combination led to a more pronounced decrease in HOMA-IR index compared to berberine monotherapy. Immunofluorescence analysis further revealed enhanced levels of insulin and glucagon in the combination group [172]. In db/db mice, this combined regimen also resulted in sustained reductions in fasting blood glucose and improved glucose tolerance [173], indicating superior efficacy in glycemic control and islet function restoration [174].

Timosaponin B2 (TB-2), a steroidal saponin with known hypoglycemic activity, lowers fasting blood glucose through multiple mechanisms. In spontaneous non-obese diabetic Goto-Kakizaki (GK) rats, the combination of TB-2 and berberine led to greater reductions in fasting blood glucose (FBG), non-fasting blood glucose (NFBG), and OGTT levels compared to either treatment alone, suggesting a synergistic antidiabetic effect [175].

Both astragalus polysaccharide (APS) and berberine have demonstrated abilities to ameliorate insulin resistance [176]. In HFD-induced mouse models, the combination of APS and berberine resulted in significantly greater reductions in fasting blood glucose and insulin resistance index compared to monotherapies, supporting the enhanced efficacy of combination therapy in improving insulin sensitivity [177].

**Table 1:** Summary of Representative Studies on Berberine Combination Therapy for Diabetes.

Drug Combinations	In Vitro and In Vivo Models	Mechanisms of Action and Efficacy	References
Ginsenoside Rb1	Insulin-resistant 3T3-L1 cells	Inhibit inflammation	[167]
	HFD/STZ-induced diabetic rat model	Improve glucose metabolism and alleviate insulin resistance	[168]
Probiotics	Patients	Reduce blood glucose levels	[20,170]
Stachyose	High-fat diet (HFD)-induced diabetic mouse model; db/db mice	Reduce blood glucose levels	[172,173]
Timosaponin B2	Spontaneously diabetic Goto-Kakizaki (GK) rats	Reduce blood glucose levels	[175]
Astragalus polysaccharide	High-fat diet (HFD)-induced diabetic mouse model	Down-regulating FOXO1 phosphorylation and PEPCK expression, and up-regulating GLUT2	[177]

## 4. Clinical Research on the Application of Berberine

In recent years, numerous clinical studies have confirmed that berberine has a significant hypoglycemic effect in the management of type 2 diabetes, and its efficacy and safety have drawn considerable attention [178]. A 12-week randomized, double-blind, placebo-controlled trial demonstrated that berberine intervention could reduce fasting insulin levels, HbA1c, and other blood glucose control indicators in patients with prediabetes [179]. Numerous clinical trials have demonstrated that berberine can effectively reduce fasting blood glucose and glycated hemoglobin levels in patients with type 2 diabetes. A 3-month randomized, double-blind, placebo-controlled trial demonstrated that patients taking berberine daily had lower HbA1c levels and fasting blood glucose levels compared to the placebo group [20]. Another randomized, double-blind, placebo-controlled, multicenter trial demonstrated that berberine monotherapy is effective and safe for treating newly diagnosed diabetes patients with dyslipidemia, resulting in significant reductions in blood glucose, blood lipids, body weight, and blood pressure within 3 months [180]. A clinical trial of berberine treatment in patients with type 2 diabetes found that monotherapy with berberine significantly reduced HbA1c levels (from 9.5% to 7.5%), fasting blood glucose (from 10.6 mmol/L to 6.9 mmol/L), and postprandial blood glucose (from 19.8 mmol/L to 11.1 mmol/L), with its efficacy comparable to that of metformin [18]. This effect was also observed in studies involving combination therapy with berberine [181]. Patients with type 2 diabetes who took berberine combined with cinnamon capsules had significantly lower fasting blood glucose (FBS) and glycated hemoglobin (HbA1c) compared to those taking placebo capsules [182]. Another study administering berberine combined with fenugreek seed capsules and placebo treatment for 12 weeks found that the intervention group containing berberine had significantly reduced fasting insulin and HbA1c levels [183]. The trial involving berberine combined with bifidobacteria also observed a decrease in fasting blood glucose levels in patients with type 2 diabetes [184].

## 5. Protective Effects and Underlying Mechanisms of Berberine Against Diabetic Complications

**Figure 4:**
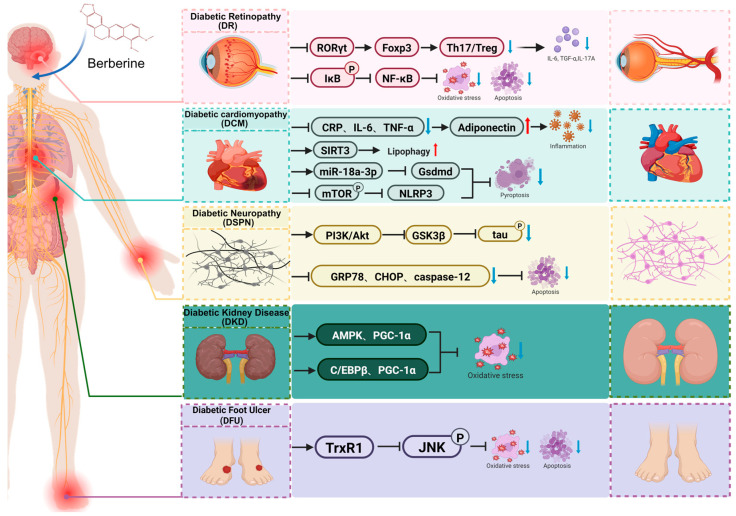
Multi-Target Mechanisms of Berberine in Preventing and Treating Multiple Diabetic Complications. Created in BioRender. tt, z. (2025) https://BioRender.com/195s48t (accessed on 11 December 2025). The arrows in the figure indicate the nature of regulation: solid black arrows represent positive regulation (activation/promotion), while black flat-headed arrows denote negative regulation (inhibition/blocking). Red flat-headed arrows specifically indicate the inhibitory effect of berberine (BBR). Blue arrows represent downregulation, and red arrows represent upregulation.

With the progression of diabetes, patients frequently develop multiple complications, which involve various organ systems—such as the cardiovascular system, nervous system, kidneys, eyes, and feet, significantly impair their quality of life and reduce life expectancy [185]. Diabetic Complications arise through complex pathogenic mechanisms closely associated with chronic hyperglycemia, insulin resistance, oxidative stress, and inflammatory responses [119]. Berberine, a natural alkaloid with diverse pharmacological properties, has recently shown considerable promise in the treatment of type 2 diabetes-related complications (Figure 4).

### 5.1. Diabetic Kidney Disease (DKD)

Diabetic Kidney Disease (DKD) represents one of the most prevalent and serious complications of diabetes, serving as a leading cause of disability and mortality among diabetic patients [186]. The pathological hallmarks of DKD include glomerulosclerosis, tubulointerstitial fibrosis, and thickening of the glomerular basement membrane [187]. The progression of DKD is closely associated with hyperglycemia-induced glomerular hyperfiltration, renal tubular epithelial cell injury, and excessive extracellular matrix accumulation [188]. Berberine has been shown to exert renal protective effects in the context of diabetic nephropathy.

In murine DKD models, berberine administration significantly reduced fasting blood glucose levels and improved key renal function parameters, such as serum creatinine, kidney-to-body weight ratio, albuminuria, urinary creatinine, and blood urea nitrogen. Moreover, berberine ameliorated pathological changes including glomerular basement membrane thickening and glomerulosclerosis, thereby attenuating glomerular fibrosis [143]. Studies conducted in HFD/STZ-induced diabetic rats and high glucose-stimulated HK-2 cells further demonstrated that berberine suppresses high glucose-induced epithelial–mesenchymal transition (EMT) and renal interstitial fibrosis by inhibiting NLRP3 inflammasome activation [189].

Excessive mitochondrial fission and mitochondrial dysfunction in podocytes have been identified as early pathological features preceding the clinical onset of diabetic kidney disease (DKD) [190]. Growing evidence indicates that berberine protects podocytes by modulating mitochondrial energy metabolism, thereby retarding DKD progression [143,191]. In palmitic acid-stimulated mouse podocytes, berberine upregulates AMPK and PGC-1α, which reduces mitochondrial fragmentation, decreases mitochondrial ROS production, and enhances ATP levels, ultimately improving mitochondrial dynamics and function [191]. Consistent with these findings, in vitro studies revealed that berberine downregulates Drp1 expression at both mRNA and protein levels, inhibits Drp1-mediated mitochondrial fission, and helps restore normal mitochondrial morphology [192]. In vivo experiments revealed that berberine-treated db/db mice exhibited reduced 24 h albumin excretion rate (AER), lowered plasma fatty acids, and decreased glomerular triglyceride (TG) content, indicating improved metabolic profile and reduced renal lipid accumulation. Additionally, berberine alleviated diabetic glomerular injury by mitigating oxidative stress and preserving slit diaphragm proteins (SDs) [191]. Other in vivo studies confirmed that berberine treatment reduces renal oxidative stress and ameliorates mitochondrial morphology in the kidneys of DKD mice [143,192].

### 5.2. Diabetic Retinopathy (DR)

Diabetic retinopathy (DR), the most common diabetic ocular complication, represents the leading cause of blindness in diabetic patients [193]. Hyperglycemia induces damage to the retinal microvasculature, leading to increased vascular permeability, pathological neovascularization, and vitreous hemorrhage. The pathogenesis of DR involves oxidative stress, inflammatory mediators, and genetic susceptibility [194]. Studies have reported that berberine can effectively ameliorate retinal injury in DR through its glucose-lowering, anti-inflammatory, and microcirculation-improving properties [195].

Both in vivo and in vitro experiments have shown that berberine significantly reduces the expression of inflammatory cytokines, including IL-6, IL-1β, TNF-α, and IL-17A. Mechanistically, berberine directly inhibits the expression of the Th17-specific transcription factor RORγt while promoting Foxp3 expression in T cells, contributing to immune homeostasis in the retina [196]. In type 2 diabetic mouse models, berberine administration improves fundus morphology and vascular networks, inhibits pathological capillary formation, and reduces the endothelial cell-to-extracellular matrix ratio, collectively ameliorating retinal endothelial dysfunction and preserving retinal integrity [197]. Additionally, berberine has been shown to inhibit NF-κB signaling by suppressing IκB phosphorylation at Ser32, thereby attenuating oxidative stress and retinal ganglion cell apoptosis in DR rat models. Consistent effects were observed in high glucose-stimulated Müller cells [198].

### 5.3. Diabetic Cardiomyopathy (DCM)

Cardiovascular disease represents one of the most prevalent and severe complications in diabetic patients, with studies confirming that diabetes significantly increases the incidence of cardiovascular disorders [199]. Insulin resistance and hyperinsulinemia contribute to dyslipidemia, characterized by elevated low-density lipoprotein cholesterol (LDL-C) and reduced high-density lipoprotein cholesterol (HDL-C), which further accelerates the progression of atherosclerosis [200].

Macrovascular complications, particularly atherosclerosis, pose a major health threat in diabetes. Both in vivo and in vitro studies have demonstrated that berberine reduces levels of inflammatory mediators such as C-reactive protein (CRP), IL-6, and TNF-α in serum and cells, while upregulating adiponectin expression, thereby attenuating systemic inflammation and exerting protective effects against diabetic macrovascular injury [133].

Diabetic cardiomyopathy (DCM), characterized by myocardial dysfunction and structural abnormalities independent of coronary artery disease or hypertension, is another major diabetic complication. Evidence indicates that excessive lipid droplet (LD) accumulation and impaired lipophagy contribute to myocardial lipotoxicity in DCM [201]. In db/db mice and palmitic acid-treated H9C2 cells, berberine upregulates SIRT3 expression, enhances LC3-II-dependent autophagosome formation, and promotes lysosomal degradation of lipid droplets, thereby mitigating myocardial lipotoxicity [202]. Additionally, berberine could ameliorate DCM by suppressing pyroptosis. It is reported that berberine upregulates miR-18a-3p, which inhibits Gsdmd-mediated pyroptosis, leading to improved cardiac function. In db/db rats and high glucose-stimulated H9C2 cells, berberine significantly reduces cardiac injury markers (cTn-I, CK-MB), attenuates myocardial fibrosis and collagen deposition, and suppresses IL-1β release [203]. Further studies reveal that berberine inhibits mTOR phosphorylation and mitochondrial ROS generation, thereby blocking NLRP3 inflammasome activation and alleviating pyroptosis, myocardial inflammation, and fibrosis [204].

### 5.4. Diabetic Neuropathy (DSPN)

Diabetic neuropathy is a prevalent chronic complication of diabetes, categorized into peripheral, autonomic, and central forms [205]. Diabetic encephalopathy—a recently recognized central neuropathy in type 2 diabetes—is characterized by cognitive decline, tau protein hyperphosphorylation, and axonal pathology. In high-fat diet/streptozotocin-induced type 2 diabetic rats, berberine treatment significantly reduced fasting blood glucose and HOMA-IR index, ameliorating systemic metabolic disturbances. At the molecular level, berberine restored PI3K/Akt signaling activity and suppressed GSK3β overactivation in the brain, leading to markedly decreased phosphorylation of tau protein at Alzheimer’s-related epitopes (Ser202 and Ser404) [99]. Further studies in a composite AD-diabetes rat model (induced by HFD, STZ, and Aβ25-35) demonstrated that berberine significantly improved cognitive performance while reducing fasting glucose and lipid levels. Mechanistically, berberine downregulated key endoplasmic reticulum stress markers—GRP78, CHOP, and caspase-12—attenuating hippocampal neuronal damage, loss of synaptophysin immunoreactivity, Aβ deposition, and neuronal apoptosis [206].

### 5.5. Diabetic Foot Ulcer (DFU)

Diabetic foot, one of the most serious chronic complications of diabetes, arises from a combination of neuropathy, vascular impairment, and infection [207]. Research suggests that Berberine holds promise for the prevention and treatment of diabetic foot by modulating blood glucose, enhancing microcirculation, and exerting anti-infective properties [208]. In HFD/STZ-induced diabetic rats and high glucose-stimulated HaCat cells, berberine was shown to target and activate TrxR1, thereby inhibiting the downstream JNK signaling pathway and accelerating wound healing [209]. In a separate study, a berberine-loaded hydrogel dressing applied topically to foot wounds in diabetic models released a metal–organic framework in response to reactive oxygen species (ROS), exerting antibacterial, anti-inflammatory, and ROS-scavenging effects that collectively promoted wound repair [210].

## 6. Discussion and Prospects

This review systematically summarizes the multi-target therapeutic potential and molecular mechanisms of berberine in the management of T2DM and its complications. Accumulating evidence demonstrates that berberine not only reduces blood glucose by modulating key processes in glucose metabolism—including promoting glycogen synthesis, inhibiting gluconeogenesis, and enhancing glucose uptake—but also fundamentally alleviates insulin resistance through precise regulation of insulin signaling pathways, AMPK-mediated energy sensing, epigenetic modifications [18,211,212,213]. Notably, the role of berberine in anti-inflammatory and antioxidant properties enable it to disrupt the “chronic inflammation–insulin resistance” cycle in T2DM, providing a solid mechanistic foundation for its preventive and therapeutic roles in diabetic complications.

Clinical trials have demonstrated that berberine can effectively improve glycemic control indicators in patients with type 2 diabetes, such as fasting blood glucose and HbA1c, indicating a clear hypoglycemic effect [20,179,180]. In addition, berberine has shown promising potential in improving parameters related to diabetic complications, including diabetic nephropathy and diabetic peripheral neuropathy. These beneficial therapeutic outcomes are attributed to berberine’s multi-target mechanisms, which involve AMPK, PPARγ, and inflammatory pathways, and its simultaneous actions on multiple organs and systems such as the liver, muscle, adipose tissue, and intestine [76,77,123,124,125,127,191], Consequently, berberine exerts comprehensive therapeutic effects on type 2 diabetes and its complications.

Despite berberine demonstrating good therapeutic effects for T2DM, its clinical translation still faces numerous challenges. The oral bioavailability of berberine is extremely low (less than 1%) [214,215,216], which might due to berberine has low water solubility and is easily degraded in the gastrointestinal tract [217,218]. Furthermore, as a substrate for P-glycoprotein efflux pumps, it forms an absorption barrier in the intestine, which reduces its bioavailability [219]. In addition, berberine undergoes intense first-pass metabolism during absorption, primarily through phase II conjugation reactions, resulting in very low plasma concentrations of the parent drug [220]. Therefore, it is essential to explore drug formulations of berberine, such as nanoformulations and self-microemulsifying drug delivery systems, to enhance its absorption and efficacyd self-microemulsifying drug delivery systems, to enhance its absorption and efficacy [161].Safety must be considered when using berberine. Common side effects include GI upset, headache, fatigue, and hypoglycemia. A trial noted about 10% of patients experienced diarrhea/constipation, typically early in treatment [18]. A meta-analysis found a 12–15% adverse event rate (mostly mild) and a <1% rate of serious events [221]. High doses increase hypoglycemia risk, especially with other diabetes drugs [222]. It is recommended to start at a low dose (e.g., 300–500 mg/day), increase gradually, and monitor organ function and glucose [18].

In summary, berberine, as a multi-target natural agent, shows considerable promise for the integrated management of T2DM and diabetic complications. Through interdisciplinary collaboration across pharmaceutical sciences, basic research, and clinical investigation, this ancient compound is poised to gain renewed relevance and offer novel therapeutic opportunities for diabetic patients.

## Data Availability

No new data were created or analyzed in this study. Data sharing is not applicable to this article.
